# Morin, a Flavonoid from Moraceae, Induces Apoptosis by Induction of BAD Protein in Human Leukemic Cells

**DOI:** 10.3390/ijms16010645

**Published:** 2014-12-30

**Authors:** Cheol Park, Won Sup Lee, Se-Il Go, Arulkumar Nagappan, Min Ho Han, Su Hyun Hong, Gon Sup Kim, Gi Young Kim, Taeg Kyu Kwon, Chung Ho Ryu, Sung Chul Shin, Yung Hyun Choi

**Affiliations:** 1 Department of Molecular Biology, College of Natural Sciences, Dongeui University, Busan 614-714, Korea; E-Mail: parkch@deu.ac.kr; 2 Department of Internal Medicine, Institute of Health Sciences, Gyeongsang National University School of Medicine, Jinju 660-702, Korea; E-Mails: gose1@hanmail.net (S.-I.G.); arulbiotechtnau@gmail.com (A.N.); 3 Department of Biochemistry, Dongeui University College of Oriental Medicine, Busan 614-052, Korea; E-Mails: alsgh0615@lycos.co.kr (M.H.H.); hongsh326@hanmail.net (S.H.H.); 4 School of Veterinary Medicine, Research Institute of Life Science, Gyeongsang National University, Jinju 660-701, Korea; E-Mail: gonskim@gnu.ac.kr; 5 Laboratory of Immunobiology, Department of Marine Life Sciences, Jeju National University, Jeju 690-756, Korea; E-Mail: immunkim@jejunu.ac.kr; 6 Department of Immunology, School of Medicine, Keimyung University, Daegu 704-701, Korea; E-Mail: kwontk@dsmc.or.kr; 7 Division of Applied Life Science (BK 21 Program), Research Institute of Life Science, Gyeongsang National University, Jinju 660-701, Korea; E-Mail: ryu@gnu.ac.kr; 8 Department of Chemistry, Research Institute of Life Science, Gyeongsang National University, Jinju 660-701, Korea; E-Mail: scshin@gnu.ac.kr; 9 Anti-Aging Research Center & Blue-Bio Industry RIC, Dongeui University, Busan 614-714, Korea

**Keywords:** morin, apoptosis, BAD, Bcl-xL, leukemia

## Abstract

Evidence suggests that phytochemicals can safely modulate cancer cell biology and induce apoptosis. Here, we investigated the anti-cancer activity of morin, a flavone originally isolated from members of the Moraceae family in human leukemic cells, focusing on apoptosis. An anti-cancer effect of morin was screened with several human leukemic cell lines. U937 cells were most sensitive to morin, where it induced caspase-dependent apoptosis in a dose-dependent manner. It also induced loss of MMP (*ΔΨ_m_*) along with cytochrome c release, down-regulated Bcl-2 protein, and up-regulated BAX proteins. The apoptotic activity of morin was significantly attenuated by Bcl-2 augmentation. In conclusion, morin induced caspase-dependent apoptosis through an intrinsic pathway by upregulating BAD proteins. In addition, Bcl-2 protein expression is also important in morin-induced apoptosis of U937 cells. This study provides evidence that morin might have anticancer properties in human leukemic cells.

## 1. Introduction

Considerable interest has been drawn to the possibility of preventing or controlling cancer using flavonoids from fruit because high intake of fruit and vegetables is associated with low incidence of cancer [1,2]. In addition, many studies suggest that phytochemicals can safely modulate cancer cell biology and induce cancer cell death [3,4]. Morin (3,5,7,2',4'-pentahydroxyflavone) is a flavone originally isolated from members of the Moraceae family. It has been reported to have some properties that regulate the inflammatory response, and halt carcinogenesis and cancer progression [5,6]. However, few studies have been conducted regarding the anti-cancer effects of morin, and the molecular mechanisms of the anti-cancer effects are poorly elucidated in human leukemic cells.

Apoptosis is an active-energy requiring process (a type I programmed cell death) which harbors a distinctive phenotype, such as blebbing, cell shrinkage, nuclear fragmentation, chromatin condensation, and chromosomal DNA fragmentation [7,8]. This has been suggested to be one of the major mechanisms of the anti-cancer effects of fruits and vegetables. Most of apoptosis triggered phytochemicals are caspase-dependent, which usually occurs through two major pathways (the intrinsic pathway and the extrinsic pathway); the former is mitochondria-mediated, and the latter death receptor-mediated. However, the mechanisms of morin-induced apoptosis in cancer cells especially of mitochondrial proteins are not fully elucidated. Therefore, we investigated the anti-cancer activity along with the mechanisms focusing on apoptosis in human leukemic cells.

## 2. Results

### 2.1. Morin Inhibited Proliferation and Induced Apoptosis of U937 Human Leukemic Cells

**Figure 1:**
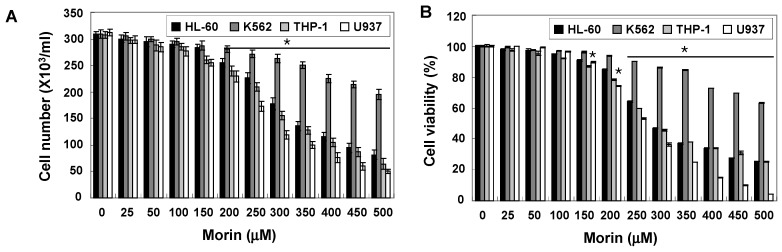

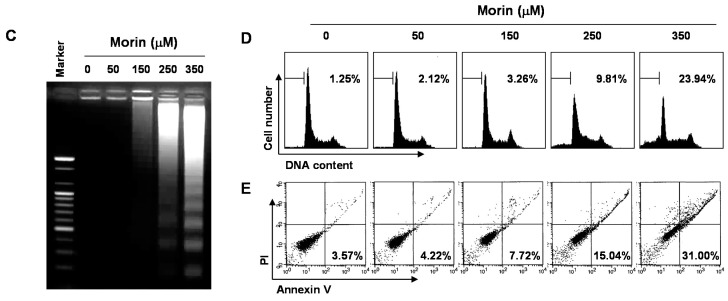
Growth inhibition and apoptosis induction by morin in U937 leukemic cells. The growth inhibition and cytotoxicity of morin are in a dose-dependent manner. U937 cells were seeded at the density of 5 × 10^4^ cells/mL. The cells were treated with indicated concentrations of morin for 48 h. (**A**,**B**) Cell viability was analyzed by (**A**) trypan blue exclusion method and (**B**) MTT assay. The data are shown as means ± SD of three independent experiments. * *p* < 0.05 *vs.* control; (**C**) DNA fragmentation test. A ladder pattern of DNA fragmentation indicates internucleosomal cleavage associated with apoptosis; (**D**) Cell cycle analysis. The cells harboring sub-G1 DNA content represents the fractions undergoing apoptotic DNA degradation by morin treatment and (**E**) Flow cytometry for the dual staining of Annexin V and PI. Annexin V^+^/PI^−^ cells indicate the cells undergoing early apoptosis. The proportion was expressed by percentage. The results are from one representative of two independent experiments that showed similar patterns.

To investigate the anti-cancer activity of morin, HL-60, K562, THP-1, and U937 human leukemic cells were treated with indicated concentrations (up to 500 μM) of morin for 48 h. A trypan blue exclusion method (Figure 1A) and an 3-(4,5-Dimethylthiazol-2-yl)-2,5-diphenyltetrazolium bromide (MTT) test (Figure 1B) revealed that U937 cells were the most sensitive to morin and K562 cells the least sensitive. The growth of U937 cells was inhibited by morin treatment in a dose-dependent manner, and IC_50_ for 48 h treatment was less than 300 μg/mL (Figure 1A,B). To investigate the mechanism of the cell death of U937 cells, we performed DNA fragmentation tests which revealed a typical ladder pattern of DNA fragmentation, which indicates internucleosomal cleavage associated with apoptosis (Figure 1C). Next, we performed cell cycle analysis to assess the population of cell death and to determine whether morin induces cell cycle arrest. As shown in Figure 1D, morin induced significant accumulation of cells with sub-G1 DNA content (apoptotic cell population) and substantially decreased the G1 fractions; in contrast, the S phase and G2M population were mildly increased. Finally we measured the early apoptotic cells (Annexin V^+^/propidium iodide (PI)^−^) by flow cytometry. The early apoptotic cells were increased in a dose-dependent manner (Figure 1E). These results suggest that the type of cell death induced by morin is apoptosis.

### 2.2. Morin Induces Caspase Activation and Subsequent Cleavage of Poly ADP Ribose Polymerase (PARP)

**Figure 2:**
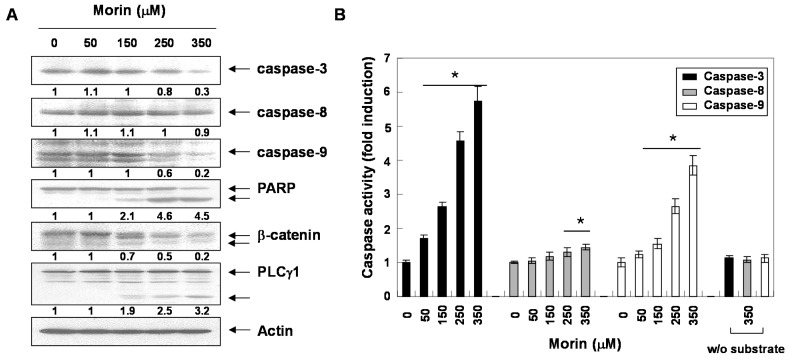
Activation of caspases and subsequent cleavage of PARP during the morin-induced apoptosis in U937 cells. U937 cells were incubated at indicated concentrations of morin for 48 h. (**A**) Western blot analysis for the effects of morin on the caspase activation and PARP cleavage. The membranes were probed with the anti-caspase-3, anti-caspase-8, anti-caspase-9 and anti-PARP antibodies. The expression of the indicated proteins were measured by densitometry and expressed as average relative ratio compared to actin, from two or three different experiments and (**B**) *In vitro* assay for caspase-3, -8 and -9 activity, which uses DEVD-pNA, IETD-pNA and LEHD-pNA as substrates, respectively. The released fluorescent products were measured. Data are expressed as mean ± SD of three independent experiments. (* *p* < 0.05 *vs.* control).

**Figure 3:**
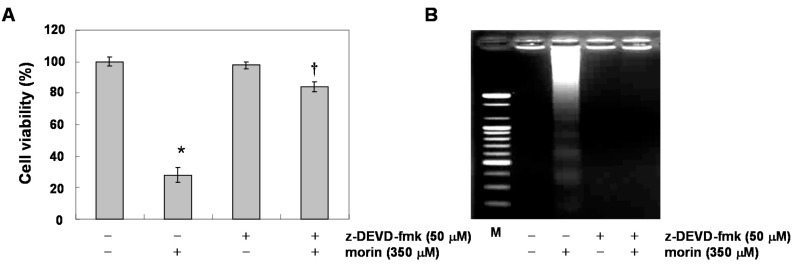

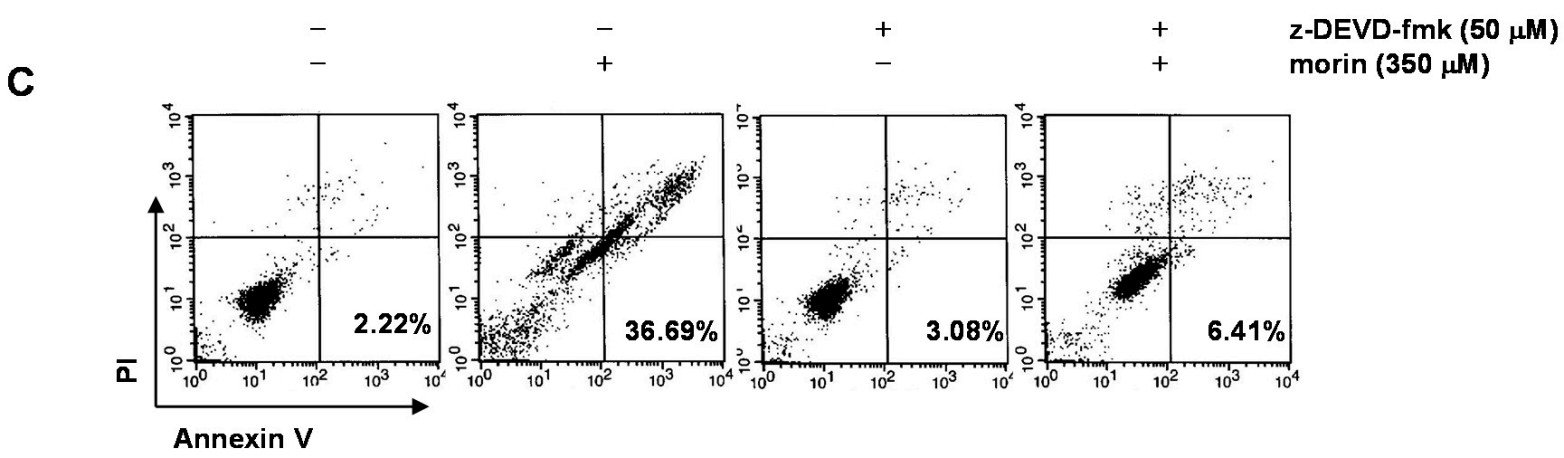
The role of caspase 3 in morin-induced apoptosis in U937 cells. The cells (5 × 10^4^ cells) were incubated at the indicated concentrations of morin with or without a caspase 3 inhibitor (z-DEVD-fmk) for 48 h. (**A**) Cell viability was assessed by MTT assay. The data are shown as means ± SD of three independent experiments. * *p* < 0.05 *vs.* control; † *p* < 0.05 *vs.* morin-treated group; (**B**) DNA fragmentation test; (**C**) Flow cytometry for the dual staining of Annexin V and PI. The proportion was expressed by percentage. The results are from one representative of two independent experiments that showed similar patterns.

Next, we determined whether morin-induced apoptosis was caspase-dependent. Western blotting analyses revealed that morin activated procaspase-3, procaspase-8, and procaspase-9 in a dose-dependent manner (Figure 2A). Morin also induced the cleavage of PARP, β-catenin, and PLCγ1 which are the substrates of caspases (Figure 2A). We next performed capase activity assays. Morin activated caspase-3 and caspase-9 rather than capase-8 in a dose-dependent manner (Figure 2B). This finding indicates that morin induced capase-3 and caspase-9, which are associated with mitochondria-mediated apoptosis. We confirmed the finding with a caspase-3 inhibitor, z**-**DEVD-fluoromethylketone (fmk). MTT and DNA fragementation assay revealed that z**-**DEVD-fmk significantly reduced morin-induced cell death (Figure 3A,B). In addition, Annexin V staining also revealed that z**-**DEVD-fmk significantly reduced morin-induced cell death Figure 3C). These findings thus suggest that morin induces caspase-dependent apoptosis.

### 2.3. Morin Induced Loss of MMP (ΔΨ_m_) Modulating Bcl-2 Family Members, BAD and Bcl-xL

**Figure 4:**
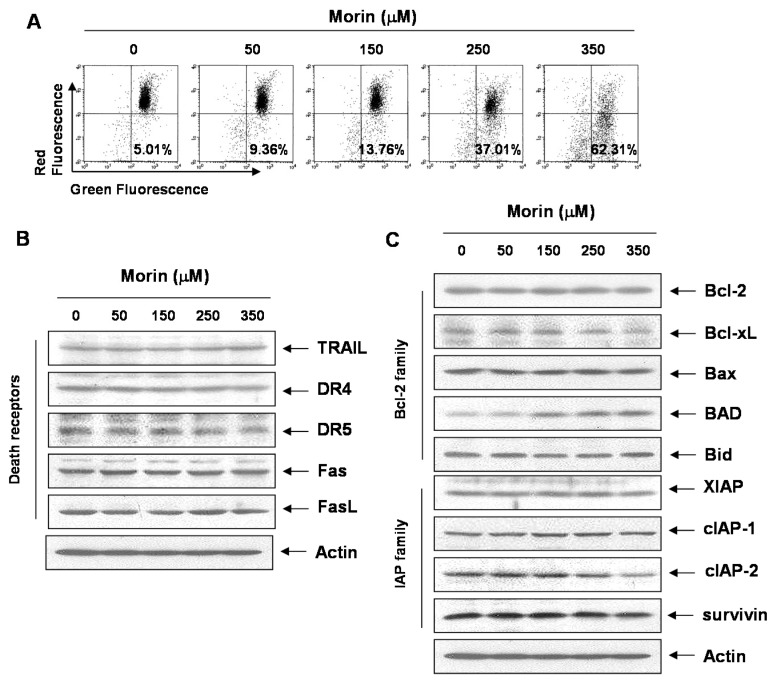
The effects of morin on mitochondrial membrane potential (*ΔΨ_m_*), and Bcl-2 family members in U937 cells. (**A**) Morin induced Loss of MMP (*ΔΨ_m_*) in a dose-dependent manner. The cells were stained with JC-1 and incubated at 37 °C for 30 min. The mean JC-1 fluorescence intensity was assessed by a flow cytometer; (**B**,**C**) The effects of morin on the expression of (**B**) death receptors (**C**) Bcl-2 and IAP family members in U937 cells. The results are from one representative of two independent experiments that showed similar patterns. The expression of the indicated proteins were measured by densitometry and expressed as average relative ratio compared to actin, from two or three different experiments.

Mitochondrial depolarization plays a critical role in apoptosis induction [9]. We measured the changes in MMP (*ΔΨ_m_*) after morin treatment. As shown in Figure 4A, morin induced the loss of MMP (*ΔΨ_m_*) in a dose-dependent manner. Next, to determine which apoptotic pathway is involved in the morin-induced apoptosis, we measured the expression of death receptors and their ligands (TRAIL receptors (DR4, DR5), TRAIL, Fas receptor (Fas), and Fas ligand (FasL)). Western blot analysis revealed that morin did not influence either death receptor expressions or their ligands (Figure 4B). We next assessed the levels of Bcl-2 and inhibitor of apoptosis (IAP) family members. Western blot analysis revealed that morin upregulated BAD protein and downregulated Bcl-xL protein (Figure 4C), suggesting that these regulatory roles are involved in morin-induced apoptosis.

### 2.4. Bcl-2 Overexpression Suppressed Morin-Induced Apoptosis and Loss of MMP (ΔΨ_m_) in U937 Cells

**Figure 5:**
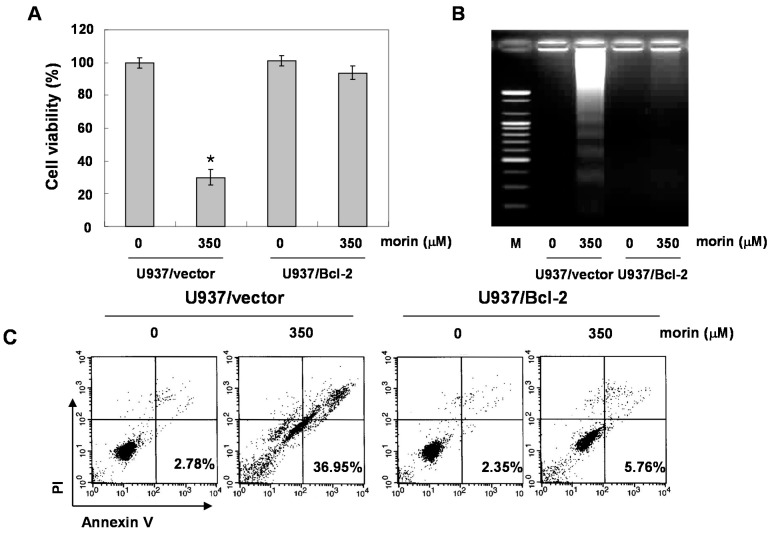

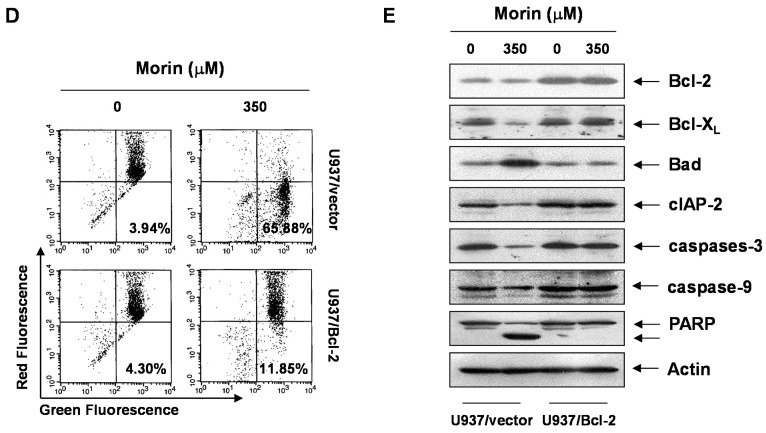
Effects of Bcl-2 overexpression on morin-induced apoptosis. U937/vector or U937/Bcl-2 cells were treated with morin for 48 h, and effects of Bcl-2 overexpression on morin-induced apoptosis. (**A**) MTT assay. The data are shown as means ± SD of three independent experiments. * *p* < 0.05 *vs.* control; (**B**) DNA fragmentation test; (**C**) Cell cycle analysis; The cells harboring sub-G1 DNA content represents the fractions undergoing apoptotic DNA degradation by morin treatment; (**D**) Flow cytometry for the dual staining of Annexin V and PI. The proportion was expressed by percentage and (**E**) The effects of Bcl-2 overexpression on the expression of Bcl-2 family members and caspases in the cells treated with morin. The expression of BAD protein was not induced in U937/Bcl-2 cells suggesting that the up-regulation of BAD expression by morin may be associated with inhibition of BAD protein degradation. The results are from one representative of two independent experiments that showed similar patterns. The expression of the indicated proteins were measured by densitometry and expressed as average relative ratio compared to actin, from two or three different experiments.

BAD forms a heterodimer with Bcl-2 and Bcl-xL, inactivating them and thus inducing apoptosis [10]. Free forms of Bcl-2 inhibits BAD-triggered apoptosis [4]. In addition, Bcl-2 is the founding member of family proteins that regulate apoptosis. Previous data suggested that BAD protein can induce apoptosis in the cells with Bcl-2 overexpression without loss of MMP (*ΔΨ_m_*) [11]. To answer the question whether morin-induced BAD can induce apoptosis without triggering the loss of MMP (*ΔΨ_m_*), we evaluated the effects of high level of Bcl-2 on morin-induced apoptosis, by comparing U937/vector with U937/Bcl-2 cells that constitutively express high levels of Bcl-2. Unexpectedly, overexpressed Bcl-2 significantly inhibited morin-induced cell death (Figure 5A), DNA fragmentation (Figure 5B), and apoptotic cell death (Figure 5C). In addition, Bcl-2 overexpression reduced morin-induced loss of MMP (*ΔΨ_m_*) (Figure 5D). Western blot analysis revealed that Bcl-2 expression level in U937/Bcl-2 cells was significantly higher (five-fold) than that in U937/vector cells, and that BAD expression was slightly lower than that in U937/vector cells (Figure 5E). The overexpressed Bcl-2 suppressed the activation of caspase 3 and subsequent PARP cleavages (Figure 5E). Interestingly, morin did not induce upregulation of BAD protein in U937/Bcl-2 cells. These finding suggests that Bcl-2 overexpression may suppress morin-induced apoptosis through inhibition of loss of MMP (*ΔΨ_m_*) and/or suppression of BAD protein expression.

## 3. Discussion

Morin (3,5,7,2',4'-pentahydroxyflavone) is a flavone originally isolated from members of the Moraceae family, such as mulberry figs, and Chinese herbs. It is also isolated as yellow pigment from almond hulls and old fustic (Chlorophora tinctoria). Morin is an isomer of quercetin (3,5,7,3,4'-pentahydroxyflavone), the lead compound for targeting phophoinositide-3-kinase (pI3K) in different cancers both* in vivo* and* in vitro* [12]. The flavonols quercetin, myricetin, and fistin, which have ortho 3',4'-di-OH in the B ring (catechol), have a stronger 2,2-diphenyl-1-picrylhydrazyl (DPPH) scavenging activity and lower inhibitory effect on superoxide production than kaempherol (4'-OH) or morin (2',4'-di-OH) [13]. In addition, morin showed similar results with apigenin (5,7,4'-trihydroxyflavone) and luteolin (5,7,3',4'-tetrahydroxyflavone) in suppression of tumor necrosis factor (TNF)-induced protein Kinase B (AKT) activation; but kaempferol (3,5,7,4'-tetrahydroxyflavone) and quercetin (3,5,7,3,4'-pentahydroxyflavone) were less active in blocking NF-κB activation [14]. However, while there is ample evidence regarding morin anti-inflammatory and anti-cancer activity, very little is known about the mechanism.

Hence, our study was designed to determine whether morin induces apoptotic cancer cell death of human leukemic cells and to further investigate the underlying mechanisms of the morin-induced apoptosis of human leukemic cells. This study demonstrates that morin induced caspase-dependent apoptosis, which was associated with BAD activation followed by loss of MMP (*ΔΨ_m_*). The maximum concentration used in the present study is two to five-fold higher than that used in many other studies showing antitumor effects of morin. It seems that the concentrations used in this study may be an obstacle for pursuing* in vivo* study. However, we previously found that the anti-cancer effects of morin are difficult to observe by MTT assay [15]. In that study, morin did not exhibit apoptotic effects up to 200 μM, but showed* in vivo* anti-cancer effects without toxicity at daily dosing with 50 mg/kg for seven days. This result supports that morin is a safe natural product with anticancer effects *in vivo.* Evidence suggested that apoptosis (type I programmed cell death) is the most popular underlying mechanism by which various anti-cancer and chemo-preventive agents including natural compounds exert anti-cancer effects [16,17,18]. Also, morin promoted ROS and Ca^2+^ productions, disruptions of mitochondria membrane potential and activated caspase-3 and caspase-9, leading to apoptosis in human leukemia HL-60 cells [19]. This study demonstrates that morin induced caspase-9, and caspase-3 activation, and the subsequent cleavages of PARP (89 kDa). Although these results suggest that morin may induce apoptosis through an intrinsic pathway, we assessed the expression of death receptors because morin has demonstrated TNF-induced apoptosis [20], and other flavonoids have already been reported to induce apoptosis by upregulating the Fas or TNF-related apoptosis-inducing ligand (TRAIL) receptors [21]. We found that morin induced apoptosis by upregulating BAD. This finding was matched with the results of caspase-9 and caspase-3 activation. For evaluation of their underlying mechanisms, we assessed Bcl-2 expression in the process of apoptosis, since Bcl-2 plays a critical role in apoptosis [9], as well as controlling BAD expression [22]. In this study, we found that overexpressed Bcl-2 suppressed morin-caspase dependent apoptosis and BAD upregulation. The anticancer activities of morin associated with Bcl-2 and X-IAP family members have been previously published [20]. These activities were mostly observed in solid cancer, not in leukemic cells. We demonstrated anti-cancer effects of morin in several leukemic cell lines and these results suggest that morin might be an effective natural compound with anti-leukemic effects. In this study we did not clearly demonstrate the mechanism of overexpressed Bcl-2-associated suppression of morin-induced BAD upregulation. In apoptosis, the ratio of Bcl-2 and BAD protein are important [23], and BAD protein is regulated by phosphorylation induced by many phosphatases and signaling pathways [22,24]. A previous study [6] suggested that morin showed anti-cancer effects by suppressing AKT activation, and we also previously demonstrated this activity [15]. MAPK and Akt activity provide well-known regulatory signals for BAD expression [18,24,25]. Another study [26] suggested that morin showed anti-cancer effects by STAT3 activity. Furthermore, we demonstrate here that Bcl-2 expression is important in morin-induced apoptosis since cells overexpressing Bcl-2 were resistant to morin therapy, suggesting that morin may not exhibit anti-cancer effects in Bcl-2-overexpressing cells. However, morin may have anti-Bcl-2 activity as shown in empty vectror transfected cells (Figure 5E). In addition, previous studies suggested that morin could inhibit PI3/Akt which regulates Bcl-2 expression [27]. Therefore, further study is warranted to elucidate the underlying mechanisms.

**Figure 6:**
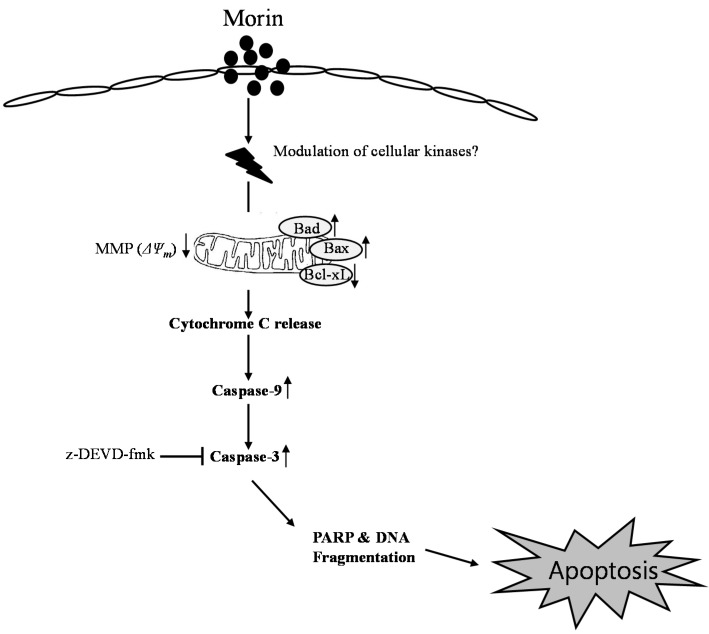
Proposed model of morin mechanism of action for apoptosis in U937 human leukemic cells. Morin induced caspase-dependent apoptosis; induced loss of MMP (*ΔΨ_m_*) along with cytochrome c release, downregulated Bcl-2 protein, and upregulated BAX proteins. It activates caspase-3 and caspase-9, and subsequent cleavage of PARP. In addition, caspase-3, z-DEVD-fmk reduced morin-induced cell death. Taken together, this study suggests that morin induces caspase-dependent apoptosis through an intrinsic pathway by modulating Bcl-2 family members, BAD and Bcl-xL, which regulates the apoptotic effect of morin in human leukemic cells.

In conclusion, morin showed anti-cancer effects in several leukemic cell lines. The cytotoxicity may result from apoptosis through the mitochondrial pathway by Bcl-2 inhibition and activation of BAD protein (Figure 6). This study provides evidence that morin might be useful for the treatment of human hematopoietic cancer cells.

## 4. Experimental Section

### 4.1. Cells and Reagents

HL-60, K562, THP-1, and U937 human leukemic cells from the American type culture collection (Manassas, VA, USA) were cultured in RPMI 1640 medium from Invitrogen (Carlsbad, CA, USA) supplemented with 10% (*v*/*v*) fetal bovine serum (FBS) from GIBCO BRL (Grand Island, NY, USA), 1 mM l-glutamine, 100 U/mL penicillin, and 100 μg/mL streptomycin at 37 °C in a humidified atmosphere of 95% air and 5% CO_2_. Morin was obtained from Aging Tissue Bank (Pusan, Korea). Antibodies against Bcl-2 (N-19), Bcl-xL, Bax, BAD, Bid, TNF-related apoptosis-inducing ligand (TRAIL), TRAIL receptors (DR4, DR5), Fas receptor, FasL, X-linked inhibitor of apoptosis protein (XIAP), cellular inhibitor of apoptosis protein-1 (cIAP-1), cIAP-2, survivin, procaspase 3, procaspase 8, procaspase 9, β-catenin, and PLCγ1 were purchased from Santa Cruz Biotechnology (Dallas, TX, USA). Antibody against poly (ADP-ribose) polymerase (PARP) was purchased from Bd Biosciences Pharmingen (San Diego, CA, USA). Peroxidase-labeled donkey anti-rabbit and sheep anti-mouse immunoglobulin, and an enhanced chemiluminescence (ECL) kit were purchased from Amersham (Waltham, MA, USA). All other chemicals not specifically cited here were purchased from Sigma-Aldrich (St. Louis, MO, USA). All these solutions were stored at −20 °C. Stock solutions of 4,6-diamidino-2-phenylindole (DAPI, 100 μg/mL) and propidium iodide (PI, 1 mg/mL) were prepared in phosphate-buffered saline (PBS).

### 4.2. Cell Viability Assay

The cell viability assay was performed using a trypan blue exclusion method and an MTT assay. For the MTT assay, cells were treated with morin for 48 h, and then incubated in 0.5 mg/mL 3-(4,5-dimethylthiazol-2-yl)-2,5-diphenyltetrazolium bromide (0.5 mg/mL) solution for 3 h at 37 °C in the dark. The absorbance of each well was measured at 540 nm with an enzyme-linked immunosorbent assay (ELISA) reader (Sunnyvale, CA, USA).

### 4.3. DNA Fragmentation Test

Cells were harvested and lysed in a buffer containing 10 mM Tris-HCl (pH 7.4), 150 mM NaCl, 5 mM EDTA, and 0.5% Triton X-100 for 1 h at room temperature. The lysates were vortexed and centrifuged at 14,000 rpm for 30 min at 4 °C. A 25:24:1 (*v*/*v*/*v*) equal volume of neutral phenol:chloroform:isoamyl alcohol was used for the extraction of the DNA from the supernatant. Electrophoretic analysis was performed on 1.5% agarose gels containing 0.1 μg/mL ethidium bromide (EtBr).

### 4.4. Flow Cytometry Analysis for Cell Cycle Analysis and Annexin V Apoptosis Assay

Cells were collected, washed with cold PBS, and then centrifuged. The pellet was fixed in 75% (*v*/*v*) ethanol for 1 h at 4 °C. The cells were washed once with PBS and resuspended in cold PI solution (50 μg/mL) containing RNase A (0.1 mg/mL) in PBS (pH 7.4) for 30 min in the dark. For annexin V double staining, the cells were collected, washed with ice cold PBS, and then centrifuged. And 5 μL of the annexin V conjugate was added to each 100 μL of cell suspension for 15 min, and 400 μL of annexin V-binding buffer was used. Flow cytometry analyses were performed with a Beckman Coulter Cytomics FC 500 (San Jose, CA, USA).

### 4.5. Measurement of Mitochondrial Membrane Potential (MMP, ΔΨ_m_) and Reactive Oxygen Species (ROS) Generation

The MMP (*ΔΨ_m_*) in living cells was measured by flow cytometry with the lipophilic cationic probe JC-1, a ratiometric, dual-emission fluorescent dye. There are two excitation wavelengths, 527 nm (green) for the monomer form and 590 nm (red) for the J-aggregate form. The cells were harvested and resuspended in 500 μL of PBS, incubated with 10 μM JC-1 for 20 min at 37 °C. Quantitation of green fluorescent signals reflects the amount of damaged mitochondria. For ROS measurement, the cells were incubated with 10 μM 2',7'-dichlorofluorescein diacetate (DCF-DA) at 37 °C for 30 min. The cells were then washed with ice-cold PBS and harvested. Fluorescence was determined by a FACS flow cytometer.

### 4.6. Western Blot Analysis

The cells were harvested and lysed. Their proteins were quantified using the BioRad protein assay (Hercules, CA, USA). For the mitochondrial fraction, we used Mitochondria Isolation Kit for Cultured Cells from Thermo Fisher Scientific (Waltham, MA USA), and followed the protocol. The final supernatant is a cytosol fraction, and the pellet contains the isolated mitochondria. The proteins of the extracts were resolved by electrophoresis, electrotransferred to a polyvinylidene difluoride membrane from Millipore (Bedford, MA, USA), and then the membrane was incubated with the primary antibodies followed by a conjugated secondary antibody to peroxidase. Blots were developed under an ECL detection system.

### 4.7. Assay of Caspase Activity

Caspase activity was measured by colorimetric assay kits, which utilized the following synthetic tetrapeptides, labeled with *p*-nitroaniline (pNA): Asp-Glu-Val-Asp (DEAD) for caspase-3, Ile-Glu-Thr-Asp (IETD) for caspase-8 and Leu-Glu-His-Asp (LEHD) for caspase-9. The cells were lysed in the supplied lysis buffer. The supernatants were collected and incubated with the supplied reaction buffer containing dithiothreitol and substrates at 37 °C. The caspase activities were determined by absorbance at 405 nm using the microplate reader.

### 4.8. Statistics

Each experiment was performed in triplicate. The results were expressed as means ± SD. Significant differences were determined using the one-way analysis of variance (ANOVA) with post-test Neuman-Keuls for the cases with at least three treatment groups, and Student’s *t*-test for two group comparison. Statistical significance was defined as *p* < 0.05.

## 5. Conclusions

In this study, we demonstrated that morin suppressed cell viability and induced caspase-dependent apoptosis in U937 cells. The induction of apoptosis was triggered through an intrinsic pathway by up-regulation of BAD proteins. In addition, Bcl-2 protein expression is also important in morin-induced apoptosis of U937 cells. This study provides evidence that morin might have anticancer properties in human leukemic cells.
